# Anaemia and Maternal Iron Intakes in Pregnancy and Child Asthma: An Observational Analysis From a Longitudinal Cohort

**DOI:** 10.1002/hsr2.72120

**Published:** 2026-03-17

**Authors:** Emma Hokey, Sophie Callanan, Anna Delahunt, Alexander Douglass, Mohammad Talaei, Seif O. Shaheen, Fionnuala M. McAuliffe

**Affiliations:** ^1^ UCD Perinatal Research Centre, School of Medicine University College Dublin, National Maternity Hospital Dublin 2 Dublin Ireland; ^2^ School of Public Health, Physiotherapy and Sports Science University College Dublin Dublin 4 Dublin Ireland; ^3^ Wolfson Institute of Population Health, Barts and The London School of Medicine and Dentistry Queen Mary University of London London UK; ^4^ Allergy and Lung Health Unit, Melbourne School of Population and Global Health The University of Melbourne Melbourne Victoria Australia

**Keywords:** childhood, dietary Iron, haemoglobin, respiratory health, ROLO

## Abstract

**Background and Aims:**

Evidence supporting the association between anaemia and lower iron intakes during pregnancy with an increased risk of childhood asthma remains unclear. This study aimed to investigate whether (i) lower haemoglobin or anaemia in late pregnancy and (ii) lower mean dietary iron intake in pregnancy were associated with a higher risk of childhood asthma. We hypothesised that lower maternal haemoglobin concentrations or anaemia, and lower mean dietary iron intakes, during pregnancy are associated with an increased risk of childhood asthma.

**Methods:**

This is a longitudinal study of mother‐child dyads (*n* = 474) who participated in the ROLO (Randomised cOntrol trial of a LOw glycaemic index diet in pregnancy to prevent macrosomia) trial between 2007 and 2022. Healthy women were recruited in early pregnancy and randomised to receive low glycaemic index dietary advice or routine care. Haemoglobin concentrations were measured in late pregnancy and anaemia was defined as < 11 g/dL. Maternal iron intakes were estimated from 3‐day food diaries during pregnancy. Mothers reported whether their children had current doctor‐diagnosed asthma at 5 and 9–11 years of age. Multivariable logistic regression models examined the associations of haemoglobin concentrations or anaemia in late pregnancy, and mean dietary iron intakes during pregnancy, with asthma outcomes.

**Results:**

In late pregnancy, the median (IQR) haemoglobin concentration was 11.7 (11.0, 12.2) g/dL and 21% (*n* = 95) of women were anaemic. The median (IQR) dietary iron intake in pregnancy was lower than recommended at 10.9 (9.3, 13.1) mg/day. The prevalence of current‐doctor diagnosed asthma at 5 and 9–11 years of age was 7% and 7.7%, respectively. Maternal haemoglobin concentrations or anaemia in late pregnancy, and mean maternal dietary iron intakes during pregnancy, were not associated with childhood asthma at either age.

**Conclusion:**

Maternal anaemia and low dietary iron intake were not associated with childhood asthma in this longitudinal study.

**Abbreviations**
ALSPAC: Avon Longitudinal Study of Adults and Children
FIGO: Federation of Gynecologists and Obstetricians
IQR: Interquartile Range
ROLO: Randomised cOntrol trial of a LOw glycaemic index diet in pregnancy to prevent macrosomia
SD: standard deviation
WHO: World Health Organisation

## 1 Introduction

Asthma is a chronic condition affecting more than 300 million people worldwide, with a large number of those affected being children [1]. The underlying origins of asthma in childhood remain unclear, but some evidence suggests that it begins during development in utero [2]. There is a growing body of interest that recognises the potential role of maternal nutrition in asthma aetiology [3, 4, 5]. Iron deficiency is the leading cause of anaemia and one of the most common nutritional deficiencies in pregnancy [6]. Reported food intakes among pregnant women have revealed that women in developed countries do not meet national dietary intake recommendations for iron, potentially leading to poor maternal and fetal health outcomes [7, 8]. A recent systematic review reported on 52 articles published between 1991 and 2021, and revealed a 36.8% overall prevalence of anaemia in pregnancies globally [9].

A number of studies have looked into the association of individual micronutrients, specific food groups, and overall diet quality with asthma outcomes in offspring [5, 10, 11]. It has been suggested that certain food types may have protective effects on asthma outcomes; the EDEN birth cohort study found favourable associations between cooked green leafy vegetable consumption before pregnancy and asthma outcomes [5].

Some observational studies have revealed associations between maternal anaemia in pregnancy and higher risk of wheeze or asthma in the offspring [12, 13]. Investigations in the Avon Longitudinal Study of Adults and Children (ALSPAC) found a weak positive association between anaemia in late pregnancy and offspring asthma at 7.5 years of age [14], while there was no association between low maternal iron status during pregnancy and offspring asthma, despite some evidence for a relation with impaired childhood lung function [15]. Further birth cohort studies reported that maternal haemoglobin concentrations were not associated with offspring asthma [16], although higher transferrin concentrations, which reflect lower serum iron concentrations during early pregnancy, were associated with higher risk of inhalant allergy, but not asthma, at 10 years of age [17]. A follow‐up analysis of a Finnish trial found routine iron supplementation throughout pregnancy was associated with a reduced risk of asthma in the offspring [18].

Plausible pathways between maternal anaemia and iron deficiency during pregnancy and childhood asthma include hypoxia, impaired immune response, and enhanced absorption of pollutant metals, which may disrupt fetal lung development and predispose children to asthma [19, 20, 21, 22, 23]. However, additional evidence is needed.

Therefore, our study aimed to examine whether lower maternal haemoglobin concentrations or anaemia, and lower mean dietary iron intakes, during pregnancy were associated with an increased risk of asthma in children who were born into the ROLO (Randomised cOntrol trial of a LOw glycaemic index diet in pregnancy to prevent macrosomia) trial.

## 2 Materials and Methods

### 2.1 Study Design and Subjects

This observational analysis used longitudinal data collected from 474 mother‐child pairs from the 5‐ and 9–11‐year follow‐up studies of the ROLO trial. The original ROLO study was a randomised control trial conducted in the National Maternity Hospital in Dublin, Ireland between 2007 and 2011. Secundigravida women (*n* = 800) who had previously delivered an infant with macrosomia (birthweight > 4 kg) were recruited in early pregnancy and randomised to receive low glycaemic index dietary advice or routine antenatal care. The primary aim of the trial was to prevent the recurrence of macrosomia. Detailed information on the study criteria, methodology and results have been previously described [24, 25]. The ROLO trial developed into a longitudinal study, with follow‐up of mother‐child dyads at 3 months, 6 months, 2 years, 5 years and 9–11 years [26, 27, 28, 29]. The 9–11 year follow‐up was completed in 2022.

The original ROLO study and the subsequent follow‐ups were carried out in accordance with the guidelines set out in the Declaration of Helsinki. Ethical approval was granted by the National Maternity Hospital Ethics Committee, Dublin, Ireland (GEN/279/12) for the primary ROLO trial. Ethical approval for the 5‐year follow‐up was obtained from the Ethics Committee (Medical Research) of Our Ladies Children's Hospital Crumlin and from University College Dublin, Office of Research Ethics Committee, Dublin, Ireland for the 9–11‐year follow‐up. The Current Controlled Trials registration number is ISRCTN54392969. Informed, written consent was obtained from the mothers prior to study participation and verbal assent was obtained from participating children.

### 2.2 Participant Selection

Participants were included in this analysis if data were available on asthma outcomes at 5 or 9–11 years of age, resulting in a total sample size of 474.

### 2.3 Exposure Assessment

#### 2.3.1 Maternal Haemoglobin Concentrations and Anaemia During Pregnancy

Maternal blood samples were taken at 13 weeks' gestation (booking visit) and at 28 weeks' gestation. Blood samples were centrifuged at 3000 rpm for 10 min and serum was obtained, sub‐aliquoted and stored at −80°C until analysis [30]. Analysis included full blood count parameters, including haemoglobin using the Sysmex XE‐2100 fluorescence flow cytometry analyser. Haemoglobin concentrations were categorised into two groups; anaemic and non‐anaemic. Anaemia was defined as a haemoglobin < 11 g/dL according to the World Health Organisation (WHO) cut‐offs [31].

#### 2.3.2 Maternal Dietary Iron Intakes During Pregnancy

At each trimester during pregnancy, dietary assessments were carried out using detailed 3‐day food diaries. The first diary was completed prior to the low glycaemic index intervention education session (10–18 weeks' gestation) and one during the second trimester (19‐27 week's gestation) and third trimester (28–35 weeks' gestation) [24]. For each record, participants were requested to fill in any food and beverages consumed over three consecutive days, which included 2 weekdays and 1 weekend day. Detailed information of portion size was also required where possible, using weights on food packaging or household measures (e.g. cups or tablespoons). Dietary data were entered into the dietary analysis software NetWISP version 3.0 (Tinuviel Software, Llanfechell, Anglesey, UK). The NetWISP software utilises the food composition database from the sixth edition of McCance and Widdowson's food composition tables, which were used to provide an estimate of the amounts and types of foods eaten and as well as the nutritional components of maternal diets [32]. Mean daily intake of macronutrients and micronutrients were generated for each timepoint in pregnancy, including iron intakes. Iron intakes were similar across trimesters, therefore, intakes in each trimester were averaged to obtain mean values through pregnancy.

### 2.4 Outcome Assessment

Of the 759 mother‐child pairs from the original ROLO study, 403 (53.0%) and 437 (57.5%) participated in the 5‐ and 9–11‐year follow‐up studies, respectively. A questionnaire was adapted from the SLAN 2007 lifestyle habits questionnaire (Survey of Lifestyle, Attitudes, and Nutrition in Ireland), which was completed by all mothers participating in the follow‐up at 5‐years and 9–11‐years about their child's health [33]. The question was described as “does your child have any ongoing problems, tick all that apply”; options listed included whether the child experienced asthma. Doctor‐diagnosed asthma was defined as the child having ongoing asthma problems that have been diagnosed by a doctor at the 5‐ and 9–11‐year follow‐ups. Asthma at 2‐years was not included in our analyses because it is difficult to distinguish asthma from preschool wheeze before the age of 5‐years [34].

### 2.5 Statistical Analysis

SPSS (Statistical Package for the Social Sciences) software version 27.0 for Mac was used to carry out all statistical analyses. Normality was tested using the Kolmogorov–Smirnov tests, along with visual inspection of histograms. Mean and standard deviation (SD) and median and interquartile range (IQR 25th, 75th percentile) were reported for normal and non‐normally distributed variables, respectively. Categorical variables with missing data were recoded to maximise statistical power by including a “missing category”. The mean imputation method was used to replace missing data for continuous variables with the mean of the available data. This method was applied to the following variables: maternal age at delivery, child age at 5‐year follow‐up, and child age at 9–11‐year follow‐up for analyses of asthma outcomes at any timepoint.

The *a priori* selection of covariates to include in exploratory adjusted analyses was informed by the literature and author consensus [35]. Covariates included original trial group (intervention/control), maternal ethnicity (White Irish, yes/no), maternal education level (three categories), smoking during pregnancy (yes/no), maternal age at delivery (years), adherence to gestational weight gain guidelines (three categories), child sex (male, yes/no), age of child at follow‐up (years), gestational age at delivery (days), and birthweight (kg). For analyses of average dietary iron intakes we additionally adjusted for mean total energy intakes during pregnancy (kcal/day) and supplement use (yes/no). Crude and adjusted multivariate logistic regression models were used to assess associations between maternal factors during pregnancy and asthma outcomes in children at any timepoint. All models were repeated in subgroup analyses to explore associations at 5 and 9–11 years of age separately. Initially, associations between maternal anaemia at any time point in pregnancy and child asthma outcomes were examined. However, due to low numbers of mothers with anaemia during early pregnancy (*n* = 6, 1.3%), we repeated the logistic regression analyses to assess associations between anaemia during late pregnancy only and asthma outcomes in children. We analysed maternal late hemoglobin concentrations and average iron intake during pregnancy in tertiles, using the lowest tertile as reference. Linear trends were tested across tertiles by allocating median values to each of the three categories which were included as a continuous variable in the models (i.e., per tertile effect). A two‐sided *p*‐value of < 0.05 was considered statistically significant for all analyses.

A sensitivity analysis was performed to examine the effect of excluding any supplement users on the associations between maternal mean dietary iron intakes during pregnancy and child asthma outcomes. Another sensitivity analysis examined the effect of excluding adjustment for potential mediators (birthweight and gestational age at delivery) in all models.

## 3 Results

### 3.1 Maternal and Child Characteristics From the ROLO Trial

The cohort characteristics for those with asthma outcomes at any timepoint are summarised in Table 1. The majority of mothers identified as White Irish (*n* = 438/474, 92.4%) and 51.7% (*n* = 245/474) had completed tertiary level education. Only 3% (*n* = 14/474) of mothers smoked in pregnancy. Half of the children were female (*n* = 239/474, 50.4%) and their median (IQR 25th, 75th percentile) age was 5.1 (5.0, 5.2) and 9.8 (9.3, 10.2) years at the 5‐ and 9–11year follow‐up, respectively. Of 474 children, *n* = 38 (8.0%) had current‐doctor diagnosed asthma at 5 or 9–11 years when both follow‐ups were combined.

**Table 1:** Maternal and child characteristics for those with asthma outcomes at any timepoint.

	Total (*N* = 474)	
	Mean/Median/*n*	SD/(IQR)/%
Maternal characteristics		
*Group*		
Intervention	237	50
Control	237	50
Age at delivery (years)	33.3	(30.5, 35.5)
HP index	7.2	(0.6, 12.1)
*Gestational weight gain*		
Inadequate, *n* (%)	65	13.7
Adequate, *n* (%)	142	30.0
Excessive, *n* (%)	186	39.2
Missing, *n* (%)	81	17.1
*Smoking in pregnancy*		
Yes, *n* (%)	14	3.0
No, *n* (%)	460	97.0
*Education level*		
Completed tertiary level, *n* (%)	245	51.7
Completed some tertiary level, *n* (%)	89	18.8
Any secondary education, *n* (%)	80	16.9
Missing, *n* (%)	60	12.7
*Ethnicity*		
White Irish, *n* (%)	438	92.4
Other, *n* (%)	36	7.6
Maternal anaemia and dietary iron intakes in pregnancy		
Haemoglobin in early pregnancy (g/dL)	12.9	(12.3, 13.5)
Anaemic, *n* (%)	6	1.3
Haemoglobin in late pregnancy (g/dL)	11.7	(11.0, 12.2)
Anaemic, *n* (%)	95	21.0
Dietary iron intake across all trimesters (mg/day)	10.9	(9.3, 13.1)
Child characteristics		
*Child sex*		
Male, *n* (%)	235	49.6
Female, *n* (%)	239	50.4
Birthweight (kg)	4.0	0.4
Gestational age at delivery (days)	283	(277, 288)
Age at 5‐year follow‐up (years)	5.1	(5.0, 5.2)
Age at 9–11‐year follow‐up (years)	9.8	(9.3, 10.2)

*Note:* Results are presented as mean (SD standard deviation) for normally distributed variables, median (IQR interquartile range 25th, 75th percentile) for non‐normally distributed variables, and *n* (%) for categorical variables.

Abbreviations: HP Index, Haase and Pratschke index; ROLO, Randomised cOntrol trial of LOw glycaemic index diet in pregnancy vs. no dietary intervention to prevent recurrence of macrosomia.

### 3.2 Maternal Dietary Iron Intakes, Haemoglobin Concentrations, and Anaemia During Pregnancy

Description of maternal dietary iron intakes, haemoglobin concentrations, and anaemia during pregnancy is provided in Table 1. Of 390 women, the median (IQR 25th, 75th percentile) dietary iron intake across all trimesters of pregnancy was 10.9 (9.3, 13.1) mg/day. In early pregnancy, the median (IQR 25th, 75th percentile) haemoglobin concentration was 12.9 (12.3, 13.5) g/dL, and 1.3% (*n* = 6/450) of women were anaemic. In late pregnancy, the median (IQR 25th, 75th percentile) haemoglobin concentration was 11.7 (11.0, 12.2) g/dL, and 21% (*n* = 95/451) of women were anaemic.

### 3.3 Associations of Maternal Anaemia and Haemoglobin in Pregnancy With Child Asthma

In crude and adjusted models, there was no association between maternal anaemia at any time point in pregnancy or in late pregnancy and childhood asthma at any age, or at 5‐years and 9–11‐years of age (Tables 2 and 3). There was no association between maternal haemoglobin concentrations in late pregnancy and childhood asthma at any age (Table 4).

**Table 2:** Associations between anaemia at any time in pregnancy and asthma outcomes in childhood.

	Non‐anaemic (Hb ≥ 11 g/dL)	Anaemic (Hb < 11 g/dL)	
	Ref	OR (95% CI)	*p* value
Asthma			
Any time point			
Cases/non‐cases [cases %]	30/343 [8.0%]	8/90 [8.1%]	0.96
Unadjusted	1.00	0.98 (0.43, 2.22)	0.96
Adjusted^a^	1.00	0.95 (0.40, 2.24)	0.91
5 years			
Cases/non‐cases [cases %]	20/255 [7.2%]	5/71 [6.5%]	0.83
Unadjusted	1.00	1.11 (0.40, 3.07)	0.83
Adjusted^a^	1.00	1.10 (0.37, 3.26)	0.86
9–11 years			
Cases/non‐cases [cases %]	25/277 [8.2%]	5/75 [6.2%]	0.54
Unadjusted	1.00	1.35 (0.50, 3.65)	0.55
Adjusted^a^	1.00	1.36 (0.47, 3.92)	0.57

*Note:* Values determined using logistic regression.

^a^ Model adjusted for child sex, age at follow‐up, trial group, maternal education, maternal ethnicity, maternal age at delivery, maternal smoking in pregnancy, gestational weight gain, birthweight, gestational age at delivery.

**Table 3:** Associations between anaemia in late pregnancy and asthma outcomes in childhood.

	Non‐anaemic (Hb ≥ 11 g/dL)	Anaemic (Hb < 11 g/dL)	
	Ref	OR (95% CI)	*p* value
Asthma			
Any time point			
Cases/non‐cases [cases %]	29/327 [8.1%]	8/87 [8.4%]	
Unadjusted	1.00	0.96 (0.42, 2.18)	0.93
Adjusted^a^	1.00	0.92 (0.39, 2.18)	0.86
5 years			
Cases/non‐cases [cases %]	19/249 [7.1%]	5/69 [6.7%]	
Unadjusted	1.00	1.05 (0.37, 2.92)	0.92
Adjusted^a^	1.00	1.01 (0.34, 3.02)	0.97
9–11 years			
Cases/non‐cases [cases %]	25/270 [8.4%]	5/72 [6.4%]	
Unadjusted	1.00	1.33 (0.49, 3.60)	0.57
Adjusted^a^	1.00	1.32 (0.45, 3.81)	0.60

*Note:* Values determined using logistic regression.

^a^ Model adjusted for child sex, age at follow‐up, trial group, maternal education, maternal ethnicity, maternal age at delivery, maternal smoking in pregnancy, gestational weight gain, birthweight, gestational age at delivery.

**Table 4:** Associations between maternal haemoglobin concentrations during late pregnancy and child asthma outcomes.

Tertiles of late haemoglobin concentrations during pregnancy (g/dL)				*p* for trend	Per Tertile	Per g/dL of haemoglobin
	T1 (8.50–11.30 g/dL)	T2 (11.31–11.99 g/dL)	T3 (12.0–14.2 g/dL)			
Asthma						
Any time point						
Cases/non‐cases [cases %]	14/154 [8.3%]	9/126 [6.6%]	14/134 [9.4%]			37/414 [8.2%]
OR (95% CI)^a^	1.00	0.78 (0.32, 1.87)	1.14 (0.52, 2.49)	0.75	1.07 (0.71, 1.60)	1.14 (0.77, 1.67)
OR (95% CI)^b^	1.00	0.79 (0.31, 1.97)	1.23 (0.54, 2.82)	0.64	1.11 (0.72, 1.70)	1.18 (0.78, 1.78)
5‐years						
Cases/non‐cases [cases %]	9/124 [6.7%]	7/88 [7.3%]	8/106 [7.0%]			24/318 [7.0%]
OR (95% CI)^a^	1.00	1.09 (0.39, 3.05)	1.04 (0.38, 2.79)	0.93	1.02 (0.62, 1.66)	1.12 (0.70, 1.80)
OR (95% CI)^b^	1.00	1.12 (0.37, 3.41)	1.02 (0.35, 2.99)	0.95	1.01 (0.59, 1.72)	1.13 (0.69, 1.86)
9–11 years						
Cases/non‐cases [cases %]	11/125 [8.1%]	6/111 [5.1%]	13/106 [10.9%]			30/342 [8.1%]
OR (95% CI)^a^	1.00	0.61 (0.22, 1.71)	1.39 (0.59, 3.24)	0.46	1.19 (0.76, 1.88)	1.27 (0.82, 1.97)
OR (95% CI)^b^	1.00	0.59 (0.19, 1.79)	1.67 (0.66, 4.24)	0.30	1.31 (0.80, 2.16)	1.41 (0.87, 2.27)

*Note:* Values determined using multivariate logistic regression.

Abbreviations: CI, confidence interval; OR, odds ratio; T, tertile.

^a^ Crude model.

^b^ Model 1 adjusted for child sex, age at follow‐up, trial group, maternal education, maternal ethnicity, maternal age at delivery, maternal smoking in pregnancy, gestational weight gain, birthweight, gestational age at delivery.

### 3.4 Associations of Mean Maternal Dietary Iron Intakes in Pregnancy With Child Asthma

In all models, there were no associations between mean maternal dietary iron intakes in pregnancy and childhood asthma outcomes at any time point, or at 5‐years and 9–11‐years of age (Table 5).

**Table 5:** Associations between maternal mean iron intakes during pregnancy and child asthma outcomes.

Tertiles of mean iron intake during pregnancy (mg/day)				*p* for trend	Per Tertile	Per mg/day of mean iron intake
	T1 (5.05–9.70 mg/day)	T2 (9.71–12.21 mg/day)	T3 (12.22–29.64 mg/day)			
Asthma						
Any time point						
Cases/non‐cases [cases %]	8/115 [6.5%]	14/123 [10.2%]	10/120 [7.7%]			32/358 [8.2%]
OR (95% CI)^a^	1.00	1.63 (0.66, 4.04)	1.19 (0.45, 3.14)	0.84	1.07 (0.68, 1.69)	1.01 (0.89, 1.14)
OR (95% CI)^b^	1.00	1.60 (0.58, 4.44)	1.07 (0.31, 3.70)	0.92	1.01 (0.56, 1.83)	0.97 (0.81, 1.16)
5‐years						
Cases/non‐cases [cases %]	8/90 [8.1%]	10/98 [9.2%]	4/99 [3.8%]			22/287 [7.1%]
OR (95% CI)^a^	1.00	1.14 (0.43, 3.03)	0.45 (0.13, 1.56)	0.19	0.71 (0.41, 1.24)	0.94 (0.79, 1.10)
OR (95% CI)^b^	1.00	0.82 (0.26, 2.59)	0.23 (0.04, 1.16)	0.06	0.51 (0.24, 1.07)	0.84 (0.66, 1.06)
9–11 years						
Cases/non‐cases [cases %]	6/100 [5.6%]	9/100 [8.2%]	9/98 [8.4%]			24/298 [7.4%]
OR (95% CI)^a^	1.00	1.50 (0.51, 4.37)	1.53 (0.52, 4.46)	0.47	1.22 (0.72, 2.04)	1.06 (0.91, 1.22)
OR (95% CI)^b^	1.00	1.60 (0.46, 5.49)	1.59 (0.39, 6.42)	0.57	1.24 (0.62, 2.45)	1.10 (0.90, 1.36)

*Note:* Values determined using multivariate logistic regression.

Abbreviations: CI, confidence interval; OR, odds ratio; T, tertile.

^a^ Crude model.

^b^ Model 1 adjusted for child sex, age at follow‐up, trial group, maternal education, maternal ethnicity, maternal age at delivery, maternal smoking in pregnancy gestational weight gain, birthweight, gestational age at delivery, supplement intake, mean energy intake.

### 3.5 Sensitivity Analyses

Associations of mean maternal dietary iron intakes in pregnancy with child asthma were similar after the exclusion of supplement users (data not shown). All adjusted analyses were repeated not controlling for potential mediators (birthweight and gestational age at delivery) and this yielded similar results (data not shown).

## 4 Discussion

### 4.1 Main Findings

We found that lower maternal haemoglobin concentrations and lower maternal iron intakes during pregnancy were not associated with a higher risk of childhood asthma in this Irish longitudinal study. However, we found that the dietary iron intake in this pregnant cohort was lower than recommended, at 10.9 mg/day across all trimesters.

Extensive research has investigated the link between pregnancy complications and adverse outcomes in children, however, limited studies have explored the associations of maternal iron status with childhood asthma. One of the first studies to investigate this topic was the ALSPAC birth cohort, which identified an inverse association between prenatal iron status, as inferred from umbilical cord tissue, and childhood wheezing and eczema in over 2000 participants [36]. Following on from this study, the authors investigated in ALSPAC whether low maternal haemoglobin concentrations in pregnancy were associated with increased risk of respiratory and allergic childhood outcomes [14]. No association with offspring asthma was found when haemoglobin was analysed as a continuous variable; however, there was some evidence for a positive association between anaemia in late pregnancy and childhood asthma at 8.5 years [14]. Our findings contrast with other observational studies that have reported a significant association of maternal haemoglobin with the development of asthma in childhood. A study of 597 mother‐child pairs in the United States found maternal anaemia measured prior to delivery was positively associated with risk of asthma at 6 years of age, although this was reported only in the children of mothers with asthma [12]. This finding was further explored in the ALSPAC birth cohort, but no evidence for effect modification by maternal asthma was found [14].

The most convincing evidence to date for a link between low prenatal iron status and increased risk of asthma comes from the follow up of a Finnish trial of iron supplementation in pregnancy [18]. This study (*n* = 2694 women) used health register linkage to ascertain asthma in the offspring between the ages of 10–32 years. Offspring of women who received 100 mg elemental iron per day throughout pregnancy, regardless of haemoglobin concentration, had a lower risk of asthma compared to offspring of women who received 50 mg of elemental iron twice daily, only if they were anaemic. The randomised design of this research strengthens causal inference, reducing the likelihood of unmeasured or residual confounding which is a problem in observational studies. Furthermore, a recently published meta‐analysis of 8 pooled studies showed that maternal anaemia during pregnancy was associated with a modest increase in childhood asthma risk (aOR 1.19, 95% CI: 1.02–1.38). This apparent small effect size may explain the inability of our study to detect an effect due to the small sample size [37].

According to European Food Safety Authority guidelines, pregnant women should be consuming approximately 16 mg of iron per day from food sources, however, the mean maternal intakes of iron in our cohort were below the population reference intake [38]. Maternal haemoglobin concentrations were low and the prevalence of anaemia in late pregnancy was marginally higher compared to findings from a recent systematic review that reported 17.2% prevalence of anaemia overall in pregnancy among European studies (*n* = 5) investigated [9].

### 4.2 Mechanisms

There are several potential underlying intrauterine mechanisms that associate maternal anaemia and iron deficiency in pregnancy with childhood asthma. Evidence from animal studies suggest that an inadequate supply of iron, resulting in hypoxia, can disrupt alveolar development and surfactant production critical for lung maturation and increase airway hyperresponsiveness [19, 20]. Additionally, maternal nutritional deficiencies of iron can impair fetal immune development (T‐helper cell differentiation and cytokine balance) resulting in a persistence of Th2 immune responses postnatally [21]. Another plausible explanation may be that prenatal exposure to maternal iron deficiency enhances the intestinal absorption of pollutant metals, such as manganese, lead and cadmium, that have been associated with an intrauterine predisposition to asthma [22, 23]. The influence of the timing of exposure is also unclear, with evidence suggesting that anaemia and iron deficiency in early versus late pregnancy can induce differential responses in fetal lung development [37].

### 4.3 Policy Implications and Future Research

Anaemia can have lasting health implications for mothers and offspring, thus it is paramount to tackle this problem in the preconception period and in early pregnancy [39]. The Federation of Gynecologists and Obstetricians (FIGO) have created a nutrition assessment tool called the FIGO Nutrition Checklist, that can be used to identify nutritional deficiencies among pregnant women and in the preconception period [40]. Going forward this tool may help identify nutritional deficiencies, with early intervention playing a key role in reducing rates of anaemia, thus potentially minimising poor maternal and child health outcomes [41]. Future randomised controlled trials of iron supplementation in iron‐deficient pregnant populations with longitudinal follow‐up of offspring are essential to further investigate the potential effects of maternal anaemia on childhood respiratory outcomes.

### 4.4 Strengths and Limitations

A particular strength of the study is the longitudinal aspect of the ROLO study, where mother and child pairs have been followed up over a 10 year period. Another strength of the study was the consistency of data collection methods at each follow‐up. Extensive dietary data were recorded at each trimester during pregnancy, using 3‐day food diaries, a more accurate method for dietary data collection than food frequency questionnaires. Haemogloblin levels were measured in early and late pregnancy and then extracted from charts, which provided comprehensive data on their haemoglobin concentrations and therefore reduced the likelihood of selection or measurement bias.

We acknowledge that the small sample size is a major limitation of this study, which is likely to have been underpowered and means that we were unable to detect associations of modest magnitude. Our use of maternal iron biomarkers was limited to maternal haemoglobin and iron intakes, with previous studies exploring additional biomarkers. Incorporation of other iron kinetic biomarkers such as ferritin and hepcidin in pregnant women and their children could allow a more comprehensive analysis of the relation between maternal iron status and child asthma. Another limitation was the considerable loss to follow‐up in the follow‐up studies. Additionally, whilst nutritional supplement information enabled us to carry out a sensitivity analysis, the supplement name was not reported, therefore it is unknown whether these supplements contained iron or not. However, we acknowledge that most common prenatal supplements would contain iron at much larger amounts beyond dietary sources.

To conclude, this study found that maternal iron intakes and maternal haemoglobin during pregnancy were not associated with childhood asthma outcomes.

## Author Contributions

**Emma Hokey:** writing – original draft, formal analysis, investigation, methodology, conceptualization. **Sophie Callanan:** writing – review and editing, methodology, supervision. **Anna Delahunt:** writing – review and editing, methodology. **Alexander Douglass:** writing – review and editing, data curation, resources. **Mohammad Talaei:** writing – review and editing. **Seif Shaheen:** writing – review and editing, conceptualization, investigation. **Fionnuala M. McAuliffe:** writing – review and editing, supervision, funding acquisition, conceptualization, methodology, investigation.

## Conflicts of Interest

The authors declare no conflicts of interest.

## Transparency Statement

The lead author Fionnuala M. McAuliffe affirms that this manuscript is an honest, accurate, and transparent account of the study being reported; that no important aspects of the study have been omitted; and that any discrepancies from the study as planned (and, if relevant, registered) have been explained.

## Data Availability

The data that support the findings of this study are available from the corresponding author upon reasonable request.
